# The identification of new roles for nicotinamide mononucleotide after spinal cord injury in mice: an RNA-seq and global gene expression study

**DOI:** 10.3389/fncel.2023.1323566

**Published:** 2023-12-14

**Authors:** Chunjia Zhang, Yan Li, Fan Bai, Zuliyaer Talifu, Han Ke, Xin Xu, Zehui Li, Wubo Liu, Yunzhu Pan, Feng Gao, Degang Yang, Xiaoxin Wang, Huayong Du, Shuang Guo, Han Gong, Liangjie Du, Yan Yu, Jianjun Li

**Affiliations:** ^1^School of Rehabilitation, Capital Medical University, Beijing, China; ^2^Department of Spinal and Neural Functional Reconstruction, China Rehabilitation Research Center, Beijing, China; ^3^China Rehabilitation Science Institute, Beijing, China; ^4^Center of Neural Injury and Repair, Beijing Institute for Brain Disorders, Beijing, China; ^5^Beijing Key Laboratory of Neural Injury and Rehabilitation, Beijing, China; ^6^School of Rehabilitation Sciences and Engineering, University of Health and Rehabilitation Sciences, Qingdao, Shandong, China; ^7^Cheeloo College of Medicine, Shandong University, Jinan, Shandong, China; ^8^Department of Orthopedics, Qilu Hospital of Shandong University, Jinan, Shandong, China

**Keywords:** spinal cord injury, nicotinamide mononucleotide, inflammation, signaling pathway, transcriptome sequencing technology

## Abstract

**Background:**

Nicotinamide mononucleotide (NMN), an important transforming precursor of nicotinamide adenine dinucleotide (NAD+). Numerous studies have confirmed the neuroprotective effects of NMN in nervous system diseases. However, its role in spinal cord injury (SCI) and the molecular mechanisms involved have yet to be fully elucidated.

**Methods:**

We established a moderate-to-severe model of SCI by contusion (70 kdyn) using a spinal cord impactor. The drug was administered immediately after surgery, and mice were intraperitoneally injected with either NMN (500 mg NMN/kg body weight per day) or an equivalent volume of saline for seven days. The central area of the spinal cord was harvested seven days after injury for the systematic analysis of global gene expression by RNA Sequencing (RNA-seq) and finally validated using qRT-PCR.

**Results:**

NMN supplementation restored NAD+ levels after SCI, promoted motor function recovery, and alleviated pain. This could potentially be associated with alterations in NAD+ dependent enzyme levels. RNA sequencing (RNA-seq) revealed that NMN can inhibit inflammation and potentially regulate signaling pathways, including interleukin-17 (IL-17), tumor necrosis factor (TNF), toll-like receptor, nod-like receptor, and chemokine signaling pathways. In addition, the construction of a protein-protein interaction (PPI) network and the screening of core genes showed that interleukin 1β (IL-1β), interferon regulatory factor 7 (IRF 7), C-X-C motif chemokine ligand 10 (Cxcl10), and other inflammationrelated factors, changed significantly after NMN treatment. qRT-PCR confirmed the inhibitory effect of NMN on inflammatory factors (IL-1β, TNF-α, IL-17A, IRF7) and chemokines (chemokine ligand 3, Cxcl10) in mice following SCI.

**Conclusion:**

The reduction of NAD+ levels after SCI can be compensated by NMN supplementation, which can significantly restore motor function and relieve pain in a mouse model. RNA-seq and qRT-PCR systematically revealed that NMN affected inflammation-related signaling pathways, including the IL-17, TNF, Toll-like receptor, NOD-like receptor and chemokine signaling pathways, by down-regulating the expression of inflammatory factors and chemokines.

## Graphical Abstract

The left side provides a text description of the key factors and pathways while the right side introduces the key factors and pathways. 
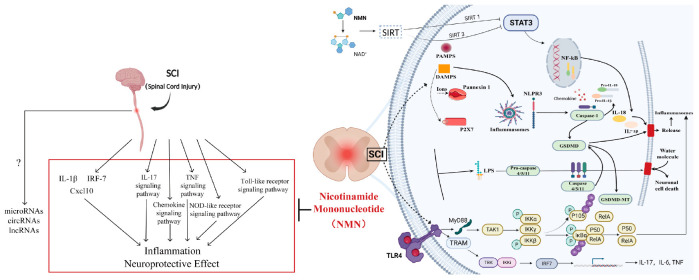

## 1 Introduction

Spinal cord injury (SCI) is a common and serious disabling disease. Most patients with SCI are young adults; traumatic SCI accounts for 90% of these cases (Alizadeh et al., 2019). The pathophysiological process of SCI can be divided into primary and secondary injury. The main cause of SCI is trauma; this mainly refers to primary mechanical injury of the spinal cord caused by direct compression or contusion of the spinal cord, accompanied by edema, hemorrhage, nerve cell apoptosis and oxidative stress; subsequently, the injury enters the secondary stage of injury (Zhu et al., 2017). Secondary inflammation after SCI is an important factor that affects prognosis and the recovery of neurological function. The destruction of nerve cells after SCI is accompanied by the infiltration of a large number of inflammatory factors. In addition, scar formation occurs at the site of injury with a prolongation in the course of disease; this seriously affects the prognosis of the disease and the recovery of neurological function. The primary mechanical injury of SCI is often inevitable. Currently, intervention measures are mainly focused on the secondary injury stage after SCI, and there is no diagnosis and treatment method that can directly exert beneficial effects on nerve regeneration and protection (Anjum et al., 2020). Therefore, it is necessary to investigate the pathophysiology and molecular mechanisms of secondary injury following SCI, to identify new intervention targets for the treatment of SCI and develop more direct and effective treatment methods.

Nicotinamide adenine dinucleotide is the core of a variety of cellular bioenergy functions, which maintains life and health and is closely related to the occurrence and development of diseases. NAD + plays an important role in basic life activities and functions, including cellular energy metabolism and cell survival (Hou et al., 2021). NAD + directly and indirectly affects many functions, including inflammatory response, metabolic pathways, DNA repair, chromatin remodeling, gene expression regulation, cell aging, cell apoptosis, and immune cell function (Hosseini et al., 2019). NMN is the most effective and direct substance used to supplement NAD + and participates in the entire process of NAD + metabolism. During organism biosynthesis, there exist two main pathways for the conversion of NMN to NAD + : one involves the catalysis of nicotinamide phosphoribosyltransferase, while the other involves remedial synthesis through the conversion of uracil nucleosides, guanine nucleosides, and nicotinamide to NAD + within the body (Burgos, 2011). The process of NMN generation is relatively intricate, but can be simplified as conversion through nicotinic acid mononucleotide (Petrelli et al., 2011). NMN serves as a crucial precursor in circulating NAD + metabolism and can boost NAD + levels *in vivo* through various pathways (Liu Y. et al., 2023). Systemic administration of NMN in animal models has been found to effectively increase NAD + levels in multiple tissues and organs (Guan et al., 2017; Martin et al., 2017), and rapid intraperitoneal injection of NMN in C57 mice has shown elevated NAD + levels in the hippocampus, indicating that NMN is capable of crossing the blood-brain barrier and promoting NAD + biosynthesis in the brain (Stein and Imai, 2014; Yoon et al., 2015). NMN administration can promote neurovascular recovery, improve mitochondrial function, have an anti-inflammatory and anti-apoptotic effect, and prevent neuronal axonal degeneration in aged mice (Kiss et al., 2020). Moreover, NMN is capable of reducing oxidative stress by decreasing CD11b infiltration into macrophages, and inhibiting photoreceptor cell death by up-regulating the expression of heme oxygenase-1 and reducing TUNEL-positively labeled photoreceptor cells, which can delay neurological inflammation and achieve overall retinal protection (Chen et al., 2020). A multicenter clinical study has confirmed the role of NMN in the treatment of disease (Yi et al., 2023). For instance, NMN can bind to the protein nucleosome assembly protein 1-like 2 and delay the aging of MSCs, which is useful for treating osteoporosis in the elderly (Hu et al., 2022). NMN also has an insulin-enhancing effect, and is able to increase insulin signaling, insulin sensitivity, and muscle remodeling in the skeletal muscle of postmenopausal post-diabetic women who are either overweight or obese when supplemented with NMN (Yoshino et al., 2021). Additionally, NMN can improve human aerobic capacity during exercise training by increasing oxygen utilization in skeletal muscle (Liao et al., 2021). In summary, the above animal experiments and clinical studies have shown that NMN has a broad array of therapeutic effects on various systemic diseases. Over recent years, NMN has gradually replaced NAD + because of its smaller molecular weight, wide range of administration methods, and high clinical conversion rate. The role of NMN in diseases of the nervous system has been gradually recognized and investigated over recent years (Kim and Yang, 2017; Hou et al., 2018; Klimova et al., 2020). This indicates that NMN may play a neuroprotective role in the prognosis of nervous system diseases and may serve as a potential therapeutic drug. However, the role of NMN in SCI has not been investigated at the molecular level; furthermore, we need to know how to apply NMN in a safe and effective manner in the clinic.

To systematically investigate the function of NMN, whole transcriptome analysis and total RNA sequencing (RNA-seq) have been performed. RNA-seq has several advantages in that it enables the detection of a wider range of coding and non-coding genes. In addition, accurate gene measurement and transcript abundance detection can be performed to better identify and understand the full characteristics of the transcriptome, thus helping us to understand the pharmacological activities of NMN more comprehensively. More importantly, RNA-seq can provide comprehensive, reliable, and detailed analysis to reveal the underlying molecular mechanisms that were not detected in previous studies.

To simulate the common types of SCI in clinical practice, we established an incomplete model of SCI in experimental mice. By examining the gene expression profile after contusion SCI in mice by whole genome sequencing, we found that the gene expression profile of mice was significantly affected after injury. SCI results in the accumulation of inflammatory factors, cell apoptosis and death at the site of injury, thus causing serious effects on nerve regeneration and repair. NMN can reduce the inflammatory response and oxidative stress caused by SCI, reduce cell apoptosis and death, and contribute to the improvement of motor function and pain threshold after SCI in mice. NMN can also regulate some typical signaling pathways. Interestingly, NMN also regulates protein expression by affecting the expression of many core genes (e.g., *IL-1*β, *IRF-7* and *Cxcl10*). In summary, RNA-seq systematically revealed the neuroprotective effects of NMN on mice after SCI through different novel mechanisms. Understanding the underlying mechanisms of action of NMN will provide us with a deeper understanding of the pharmacological activities of NMN, thus helping in the treatment of clinical patients and the development of practice guidelines. In addition, the high-throughput data collection and analysis of SCI and Sham mice in this study will allow us to better identify potential biomarkers for SCI, thus helping in the clinical diagnosis and prognosis of this disease.

## 2 Materials and methods

### 2.1 Animals, NMN, and experimental groupings

The study was conducted in accordance with the principles of the Basel Declaration and the recommendations of the National Institutes of Health Guide for the Care and Use of Laboratory Animals (NIH Publication No. 8023, revised 1978). The animal protocol was approved by the Animal Care and Use Committee of Capital Medical University (Ethics batch No. AEEI-2023-104). Adult female C57BL/6N (18–22 g) mice (8–10 weeks) were purchased from Beijing Vitong Lihua Laboratory Animal Technology Co., Ltd. (Beijing, China). Mice were maintained under standard conditions (temperature, 22 ± 2°C; Humidity, 55 ± 10%) with a 12:12 light/dark cycle. Mice were fed in a standard laboratory animal room with food and water available *ad libitum*. Adaptive feeding and training was performed for 1 week before surgery. The drug was administered immediately after surgery, and mice were injected with either NMN (500 mg NMN/kg body weight intraperitoneally per day) or an equivalent volume of saline at a fixed daily time point of 4 PM for 7 days. Twenty-four mice were randomly divided into four groups by a random number table method: a Sham group, a Sham surgery control group (*n* = 6) which underwent laminectomy without contusion; a Sham + NMN group, a Sham surgery control group (*n* = 6) which only underwent laminectomy without contusion and received NMN immediately after surgery for seven consecutive days; an SCI group, a spinal cord injury group (*n* = 6) which underwent laminectomy and contusion and received saline immediately after surgery for seven consecutive days; and an SCI + NMN group, a spinal cord injury group + NMN treatment group (*n* = 6) which underwent laminectomy and contusion and received NMN immediately after surgery for seven consecutive days. The researcher performing the surgery was unaware of the experimental groupings. Before and after surgery, behavioral tests were performed on the four groups of mice by two experienced researchers using the Basso Mouse Scale (BMS), which is often used to observe the motor function of mice (Basso et al., 2006). The BMS score includes a primary score and a secondary score. The primary score ranged from 0 (hind limb inactivity) to 9 (normal activity) while the secondary score ranged from 0 (hind limb inactivity) to 11 (normal activity), and the final score was given by consensus.

### 2.2 Mechanical allodynia and thermal hyperalgesia

The Von-Frey filament test (Aesthesio, RWD, Danmic/USA) was used to investigate for mechanical allodynia in mice after SCI. Investigators were blinded to the experimental groupings and treatments. Prior to behavioral testing, each mouse was placed in a clear glass box with an elevated wire mesh grid and acclimated to the testing cage for 30 min. Then, mechanical thresholds were measured by applying a Von-Frey wire (0.04–2.0 g) to the lateral plantar surface of each hind paw. The left and right hind limbs were tested six times with a 5-min interval between each test, and all values were averaged. To minimize stress to the animal, the thermal injury threshold was tested 1 day after the Von-Frey filament test. After the mice acclimated to the test cage for 30 min, the thermal injury threshold was assessed by the Hargreaves method of paw contraction latency (PWL) recorded with a plantar sting-machine (II TC Life Science Model 390, USA). The device was adjusted to radiate the heat source to obtain the baseline withdrawal latency of the animals (approximately 10 s). The cutoff latency was set to 15 s to avoid scalding. A 40 w infrared heat source was located below the mid-plantar area of the hind paw through the heat conduction plate, and for each test we recorded the time from stimulus onset to paw retraction. The left and right hind limbs were tested six times with a 5-min interval between each test, and all values were averaged to record nociceptive latency.

### 2.3 Establishment and sampling of the spinal cord injury model

The Spinal Cord Injury Model: Mice were anesthetized with 2% isopentane. After anesthesia, the T10 spinal cord was exposed by laminectomy. A moderate to severe spinal cord injury model was established by contusion with a spinal cord impactor (Precision Systems and Instruments IH spinal cord impactor, USA) at 70 kdyn. During the surgical procedure and recovery from anesthesia, the mice were placed in incubators until they were fully awake. After surgery, the mice were hydrated with 0.5 ml Ringer’s solution for 3 days, supplemented with electrolytes by intraperitoneal injection to maintain fluid circulation. In addition, a daily intramuscular injection of penicillin (20000 IU) was given to each mouse for 3 days to prevent systemic infection. The mice were not restricted in terms of drinking and diet. The bladder was emptied manually at least twice a day for 7 days after surgery. Animals were euthanized if they lost > 20% of their body weight, their body temperature was below 35°C, or if they exhibited self-harm (the mouse carbon dioxide euthanasia box). Surgical interventions and postoperative animal care were performed according to the surgical guidelines and policies for rodent survival provided by the Laboratory Animal Committee of Capital Medical University. None of the experimental mice died during this study.

Tissue Sampling: All mice were anesthetized with 0.25 ml of 1% pentobarbital sodium. Samples were harvested from the central area of the spinal cord injury at 7 days after surgery and stored temporarily in medical liquid nitrogen before being placed into a refrigerator at −80°C. The tissue samples used for qRT-PCR were immediately stored in RNAsolid tissue RNA stable preservation solution (G3019, Servicebio, Wuhan, China) at 4°C overnight and then stored in a refrigerator at −80°C to prevent degradation. After harvesting, the samples were used for whole transcriptome sequencing and qRT-PCR.

### 2.4 qRT-PCR analysis

Total RNA was extracted from spinal cord tissue using the RNeasy Mini Kit (Qiagen, Valencia, CA, USA) according to the manufacturer’s instructions prior to formal quantitative real-time PCR (qRT-PCR) experiments. The reverse synthesis of cDNA by M-MLV (Takara, Japan) in a reaction system containing RNase inhibitors and a random hexamer (Takara, Japan) was then performed using a real-time PCR instrument (TianLong, gentier96r). The comparative Ct value method (ΔΔCt value method) was used for data analysis. For each sample, data were normalized to the housekeeping gene *GAPDH*. Primer sequences are shown in Supplementary Table 1. Amplification curves for candidate inflammatory factors and *GAPDH* are shown in Supplementary Figure 1. Melt curve plots for candidate inflammatory factors and *GAPDH* are shown in Supplementary Figure 2. Melt peak curve plots of candidate inflammatory factors and *GAPDH* are shown in Supplementary Figure 3.

### 2.5 RNA extraction

RNA extraction was performed by Annoroad Gene Technology (Beijing) Co., Ltd. Total RNA was extracted from spinal cord tissue using the RNeasy Mini Kit (Qiagen, Valencia, CA, USA). Total RNA samples were tested for purity using a NanoPhotometer^®^ spectrophotometer (Thermo Fisher, USA) and an Agilent 2100 RNA Nano 6000 Assay Kit (Agilent Technologies, Inc., Inc., CA, USA) to check the integrity and concentration of RNA samples.

### 2.6 Preparation of transcriptome sequencing libraries

Library preparation and sequence analysis were performed by Annoroad Gene Technology (Beijing) Co., Ltd. Three groups were included: the Sham group, the SCI group, and the SCI + NMN group (three replicates per group); 1 ng/μl was used for RNA sample preparation per sample. After the quality of total RNA samples were qualified, they were purified according to the characteristics of mRNA from different sources. Fragmentation buffer was added to the purified mRNA to produce short fragments. First strand cDNA synthesis was performed by reverse transcription with random primers. Second strand cDNA was synthesized by adding 2nd Strand Marking Buffer and 2nd strand/End Repair Enzyme Mix (Zhaoyi Century Technology Co., Ltd., Beijing, China). PCR amplification was then performed to complete library preparation. After the library had been constructed, Qubit 3.0 software (Thermo Fisher, USA) was used for preliminary quantification, the library was diluted to 1 ng/μl, and then the Agilent 2100 was used to detect the sizes of inserts in the library. The concentration of the library was accurately quantified (the effective concentration of the library was > 10 nM) to ensure the library was of good quality. Qualified libraries were then sequenced on the Illumina platform using the PE150 sequencing strategy. The RNA-seq raw data has been uploaded. All data have been successfully deposited in the NCBI Sequence Read Archive (SRA).^1^ BioProject: PRJNA977966.

### 2.7 RNA-seq data analysis

All RNA-seq data analysis was performed with the assistance of Shanghai Ouyi Biomedical Technology Co., Ltd., and incorporated the following four steps.

(1) Data Filtering and Quality Control: Illumina high-throughput sequencing results were initially in a raw format. Image data files were converted into sequenced reads (raw reads) after sequence base identification. To ensure the quality of information analysis data, the raw sequence was filtered to obtain high-quality clean reads for subsequent analysis. All downstream analyses were based on clean reads.
(2) Mouse Reference Genome Map: Sample filtered sequencing sequences were then compared with reference genes. Groups were aligned and mapped to the genome. HISAT2 (v2.1.0)^2^ was used to convert the reference genome into an index using a modified BWT algorithm.
(3) Gene Expression Estimation: RNA-seq analysis was performed by mapping.
  to genomic regions. Sequences (reads) of domains or exons were counted to estimate gene expression levels. FPKM (Fragments per Kilobase per Million Mapped Fragments) was used to quantitatively estimate gene expression values to make the estimated gene expression levels of different genes and different experiments comparable.
(4) Differentially Expressed Genes Analysis: The DESeq2 software package was used to analyze the three groups; three biological replicates were used for differential gene expression analysis (Wang et al., 2010). For gene sequencing, fold change values and q (adjusted *p*-values following correction) were used as related indicators; normally, we used a | log2 fold change| ≥ 1 and *p* < 0.05 to identify significant differences in gene expression. According to the expression level of differentially expressed transcription factors in each sample (the FPKM value), we then performed hierarchical clustering. Based on R language and Python, the ggplot system was used to identify overall clustering results for each sample.

### 2.8 Functional enrichment analysis

Differentially expressed genes (DEGs) were counted and annotated using Cluster Profiler in the R software package, Gene Ontology analysis (GO analysis), Kyoto Encyclopedia of Genes and Genomes analysis (KEGG analysis), and PPI network. A *P*-value < 0.05 identified a GO option that was significantly enriched for differentially expressed genes, this allowed us to acquire detailed information relating to DEGs. The screening of differentially expressed genes and pathways by KEGG pathway analysis allowed us to further simplify the results. The functionality of DEGs was then investigated by protein interaction network analysis (STRING).^3^

### 2.9 Data availability

Details relating to the availability of RNA-seq data are given in Table 1.

**TABLE 1:** Summary of RNA-seq data for the three experimental groups (Sham group, SCI group, and SCI + NMN group, *n* = 3).

Sample name	Number of raw reads	Raw bases	Clean reads	Clean bases	% of Clean reads
S-Sl	56.283M	8.442G	54.958M	8.244G	97.65
S-S2	61.982M	9.297G	60.312M	9.047G	97.31
S-S3	64.172M	9.626G	62.984M	9.448G	98.15
T1-L11	63.729M	9.559G	62.290M	9.344G	97.74
T1-L12	64.829M	9.724G	63.694M	9.554G	98.25
T1-L13	63.846M	9.577G	62.435M	9.365G	97.79
T2-T21	55.560M	8.334G	54.261M	8.139G	97.66
T2-T22	65.031M	9.755G	63.457M	9.519G	97.58
T2-T23	60.726M	9.109G	59.193M	8.879G	97.48

S-S1, S-S2, S-S3 represent the Sham group; T1-L11, T1-L12, T1-L13 represent the SCI group; T2-T21, T2-T22, T2-T23 represent the SCI + NMN group.

### 2.10 Statistical analyses

Statistical analysis was performed using SPSS software (version 21.0, Chicago, IL, United States). The results were shown as the mean ± SEM. Behavioral assays in different groups were analyzed by repeated measurement analysis of variance (ANOVA) followed by Tukey’s *post-hoc* test. One-way ANOVA followed by the Bonferroni’s *post-hoc* test was used for the other multiple comparisons in this study, and unpaired 2-tailed Student’s t tests were used for two-group comparisons. When *p* < 0.05, the differences were considered to show statistical significance.

## 3 Results

### 3.1 Global analysis of gene expression profiling in the mouse spinal cord after SCI and NMN treatment

**FIGURE 1:**
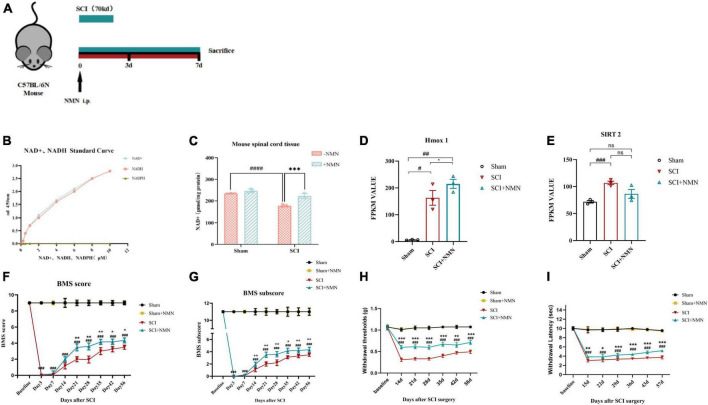
Establishment of the experimental model and mouse behavioral assessment. **(A)** One week before the establishment of the experimental animal model, environmental adaptation was performed and a moderate contusion force (70 kd) was applied. The drug was administered immediately after surgery and continuously for 7 days, and samples for analysis were taken immediately after 7 days. **(B)** A standard curve showing NAD + /NADH levels and OD values at 450 nm. **(C)** NAD + levels in mouse spinal cord tissue measured 7 days after surgery. ^####^indicates that the intergroup difference was significant when compared with the Sham group (*P* < 0.0001), ***indicates that the intergroup difference was significant when compared with the SCI group (*P* < 0.001). **(D)** The FPKM value of Hmox1 in mouse spinal cord tissue at postoperative day 7, as determined by RNA-seq. ^##^indicates statistical significance in the intergroup differences compared to the Sham group (*P* < 0.01); ^#^indicates statistical significance in the intergroup differences compared to the Sham group (*P* < 0.05); *indicates statistical significance in the intergroup differences compared to the SCI group (*P* < 0.05). **(E)** The FPKM value of SIRT2 in mouse spinal cord tissue at postoperative day 7, as determined by RNA-seq. ^###^indicates statistical significance in the intergroup differences compared to the Sham group (*P* < 0.001); ns indicates no statistical difference in the intergroup differences. **(F,G)** Experimental animal motor function score of the four groups (each group; *n* = 6 mice) at baseline, 3 days after surgery, and 1–8 weeks after surgery including BMS main score and BMS sub-score. **(H,I)** Experimental animal neuropathic pain assessment of the four groups (each group, *n* = 6 mice) at baseline, 2–8 weeks after surgery by Von-Frey mechanical pain threshold and thermal nociceptive threshold.^ #^indicates that there was a significant difference between groups when compared with the Sham group, *indicates that there was a significant difference between groups when compared with the SCI group. One, two, three, and four symbols (# or *) indicate *P* < 0.05, *P* < 0.01, *P* < 0.001 and *P* < 0.0001, respectively; ns indicates no statistical difference between groups. The Sham group, Sham + NMN group, SCI group, and SCI + NMN groups were subjected to one-way ANOVA and Bonferroni’s *post-hoc* tests.

To investigate the effect of NMN on SCI, we established a mouse model of contusion incomplete spinal cord injury (Figure 1A) and found that the levels of NAD + were significantly reduced after SCI but could be improved by supplementation with NMN (Figures 1B, C). The statistical results verified that the content of NAD + in the spinal cord tissue of mice was significantly different between SCI group and Sham group (*P* < 0.0001). The difference between SCI + NMN and SCI group was statistically significant (*P* = 0.0005, *P* < 0.001). After spinal cord injury in mice, the NAD + content in spinal cord tissue decreases, and this deficiency can be compensated by supplementation with NMN. This phenomenon may be related to changes in the levels of NAD + -dependent enzymes. Through RNA-seq screening, we identified two NAD + -dependent enzymes, Heme oxygenase-1 (Hmox-1) and Silent Information Regulator 2 (SIRT2), whose FPKM value differed among the three groups of mice (Figures 1D, E). Statistical analysis showed that in Figure 1D, the FPKM value level of Hmox-1 (FPKM value) had statistical significance between the SCI group and Sham group mice (*P* = 0.0282, *P* < 0.05), between the SCI + NMN group and Sham group mice (*P* = 0.0062, *P* < 0.01), and between the SCI + NMN group and SCI group mice (*P* = 0.046, *P* < 0.05). In Figure 1E, the FPKM value level of SIRT2 (FPKM value) had statistical significance between the SCI group and Sham group mice (*P* = 0.0007, *P* < 0.001), while there was no statistical significance in the FPKM value levels between the SCI + NMN group and Sham group mice, as well as between the SCI + NMN group and SCI group mice. Furthermore, NMN supplementation improved the motor function score (BMS score and BMS subscore) after SCI. There was a significant difference in BMS scores from day 21 after injury to the end of the experiment (Figure 1F). There was a significant difference in BMS subscore from day 14 after injury to the end of the experiment (Figure 1G). The statistical results verified that there were significant differences in BMS score and BMS subscore between SCI group and Sham group (*P* < 0.001). Compared with SCI + NMN group, BMS score was significantly different from day 21 and continued to the end of the experiment (*P*-values at each time point after spinal cord injury: 21d, *P* = 0.006; 28d, *P* = 0.003; 35d, *P* = 0.005; 42d, *P* = 0.021; 56d, *P* = 0.023) (*P* < 0.01, *P* < 0.05), and BMS subscore was significantly different from day 14 and continued to the end of the experiment (*P*-values at each time point after spinal cord injury: 14d, *P* = 0.006; 21d, *P* = 0.001; 28d, *P* = 0.003; 35d, *P* = 0.019; 42d, *P* = 0.009; 56d, *P* = 0.006) (*P* < 0.01, *P* < 0.05). In addition, NMN intervention alleviated neuropathic pain (reduced Von-Frey mechanical pain threshold and thermal nociceptive threshold) after SCI; this improvement was significantly different from days 14 and 15 after injury, respectively (Figures 1H, I). The statistical results verified that the scores of Von-Frey mechanical pain threshold and thermal nociceptive threshold in SCI group were significantly different from those in Sham group (*P* < 0.001). Compared with SCI + NMN group, Von-Frey mechanical pain threshold scores were significantly different from day 14 and continued to the end of the experiment (*P* < 0.001, *P* < 0.01), and thermal nociceptive threshold scores were significantly different from day 15 and continued to the end of the experiment (*P* < 0.001, *P* < 0.01, *P* < 0.05).

**FIGURE 2:**
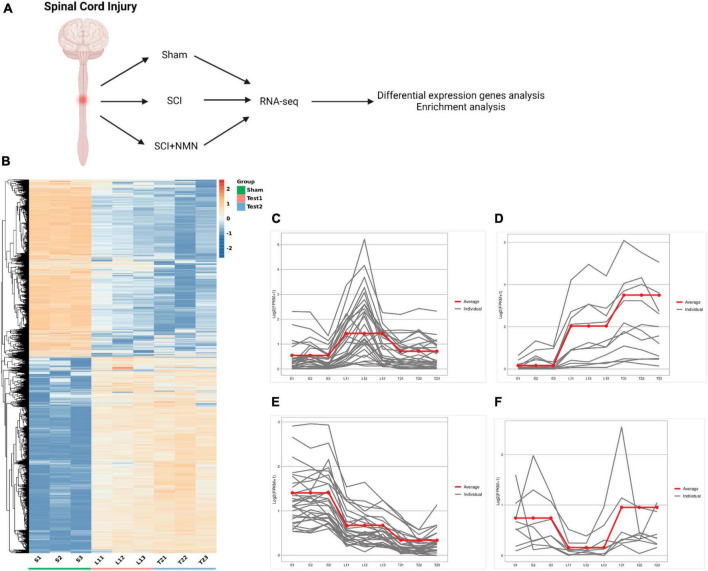
Overall analysis of gene expression profiles in mouse spinal cord after spinal cord injury and NMN treatment. **(A)** RNA-seq strategy to study the neuroprotective effects of NMN on mice with SCI. SCI, spinal cord injury. NMN, Nicotinamide Mononucleotide. **(B)** Heatmap of RNA-seq data showing the expression levels of genes in different conditions. Three biological replicates were performed for each group (repeat 1, 2 and 3). The Sham group is denoted as S. S-S1, S-S2, S-S3 represent the Sham group. The SCI group is denoted as Test 1. T1-L11, T1-L12, T1-L13 represent the SCI group. The SCI + NMN group is denoted as Test 2. T2-T21, T2-T22, T2-T23 represent the SCI + NMN group. **(C–F)** K-means clustering of gene expression profiles in different conditions. The Y-axis indicates log transformed fold-changes based on SCI condition. Three biological replicates were performed (repeat 1, 2 and 3). The gray lines show the RNA expression levels of each individual gene. The red line shows the mean RNA expression level of many individual genes. These genes could be divided into four clusters based on their expression profiles in Sham, SCI and SCI + NMN conditions.

To investigate the potential mechanism of NMN supplementation on the improvement of motor function after SCI in mice, RNA-seq was used to systematically study the mechanisms of NMN in relation to the improvement of motor function after SCI. RNA-Seq was performed on a total of nine samples from three experimental groups (Sham group, SCI group, SCI + NMN group). The total number of raw readings ranged from 55 to 65 million. After removing linker contamination, low-quality sequences, and readings containing more than 5% N, we obtained clean 54.96, 60.31, 62.98, 62.29, 63.69, 62.44, 54.26, 63.46, and 59.19 million clean readings for the nine samples (Table 1). We systematically investigated the mechanisms underlying the neuroprotective effects of NMN on SCI mice by applying RNA-seq (Figure 2A). We identified significant changes in the gene expression profiles of mice after SCI or when treated with NMN under the same injury conditions (Figure 2B). To understand how genes were affected, we performed further analysis of gene expression patterns. Genes regulated by SCI or NMN could be divided into four clusters (Figures 2C–F). Specifically, genes in cluster 1 were upregulated after SCI but significantly inhibited by NMN (Figure 2C), thus suggesting that these genes may be detrimental to mice after SCI. Genes in cluster 2 were upregulated in SCI and expressed at higher levels with NMN supplementation (Figure 2D). Compared to cluster 2 genes, the genes in cluster 3 had a completely different pattern of expression (Figure 2E). The expression levels of genes repressed by SCI in cluster 4 were significantly increased after NMN treatment, thus suggesting that these genes may be involved in the effects of NMN in mice with SCI (Figure 2F). Statistical results using | log2 fold change| ≥ 1 and *p* < 0.05 to identify significant differences in gene expression.

### 3.2 Effects of SCI on global gene expression patterns

**FIGURE 3:**
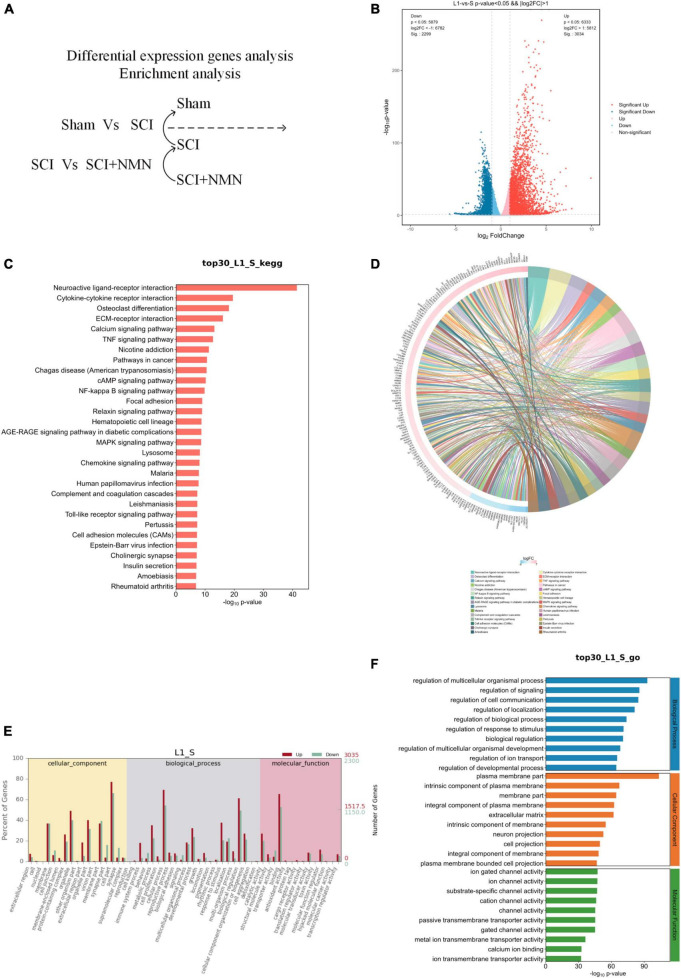
Functional enrichment analysis of genes with differential expression. S denotes Sham group, L1 denotes SCI group. **(A)** Analysis of differentially expressed genes between the SCI and Sham groups. **(B)** Analysis of differentially expressed genes. SCI upregulated or downregulated the expression levels of many genes when compared to the Sham group (SCI vs. Sham). Red dots show the genes that were upregulated by SCI while blue dots show genes that were downregulated by SCI. **(C)** Bar chart showing the top 30 genes exhibiting changes in gene expression in the SCI and Sham groups (SCI vs. Sham); these genes are involved in regulating many typical signaling pathways. **(D)** A chord diagram showing that the top 30 genes showing changes in the SCI and Sham groups (SCI vs. Sham) are involved in regulating many typical signaling pathways. **(E,F)** Gene ontology (GO) analysis of the top 30 genes in the SCI group (SCI vs. Sham group).

**FIGURE 4:**
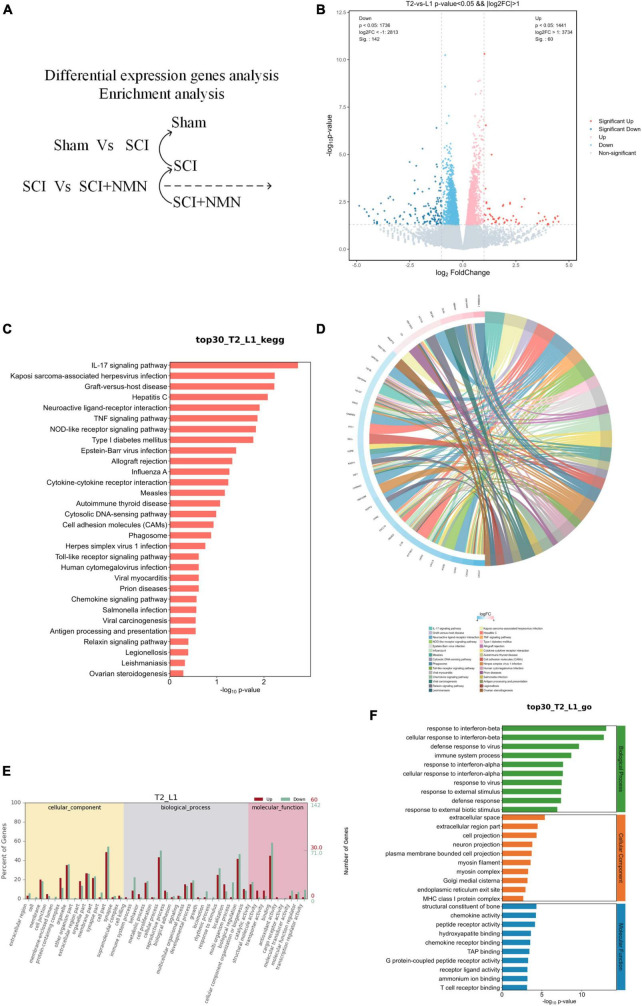
Functional enrichment analysis of genes that were differentially expressed in response to NMN treatment. L1 denotes SCI group, T2 denotes SCI + NMN group. **(A)** Analysis of differentially expressed genes between the SCI + NMN and SCI groups. **(B)** Analysis of differentially expressed genes. SCI + NMN upregulated or downregulated the expression levels of many genes when compared to that of SCI (SCI + NMN vs. SCI). Red dots show the genes that were upregulated by SCI + NMN while blue dots show the genes that were downregulated by SCI. **(C)** Bar chart showing that the top 30 genes with changes in the SCI + NMN and SCI groups (SCI + NMN vs. SCI) are involved in regulating many typical signaling pathways. **(D)** Chord diagram showing that the top 30 genes with changes in the SCI + NMN and SCI groups (SCI + NMN vs. SCI) are involved in regulating many typical signaling pathways. **(E,F)** Gene ontology (GO) analysis of the top 30 genes in the SCI + NMN group (SCI + NMN vs. SCI group).

We compared the differences in gene expression patterns between normal and SCI mice (Sham group vs. SCI group), SCI mice, and mice treated with NMN after SCI (SCI group vs. SCI + NMN group) (Figures 3A, 4A). Many genes with differential expression levels were identified in the SCI group when compared with the Sham group, including both down-regulated and up-regulated genes (Figure 3B). When compared with the SCI group, the down-regulated and up-regulated genes in the SCI + NMN group may represent target genes for subsequent experimental studies; the key molecules in these genes that require special attention are scattered on the left and right sides of the volcano plot shown in Figure 4B.

To investigate the effect of SCI, we performed functional enrichment analysis. The genes that exhibited changes when compared between the SCI and Sham groups were involved in regulating several typical signaling pathways, including neuroactive ligand-receptor interactions, and the TNF, NF-kappa B, MAPK, Chemokine, and Toll-like receptor signaling pathways (Figures 3C, D).

In addition, gene ontology (GO) analysis found that gene changes caused by SCI were significantly correlated with pro-inflammatory response and nerve aplasia, thus confirming that the inflammatory response caused by SCI was significantly correlated with nerve aplasia and neuroprotection inhibition, thus confirming that SCI promoted an inflammatory response and inhibited nerve regeneration and repair, thus affecting nerve function (Figures 3E, F). These results showed that the inflammatory response after SCI is aggravated by changes in gene expression levels which influence nerve regeneration and repair. Statistical results using fold change ≥ 2 and *p* < 0.05 to identify significant differences in gene expression.

### 3.3 NMN exerted neuroprotective effects and inhibited inflammatory response by regulating gene expression levels

To investigate the anti-inflammatory effects of NMN in SCI, we compared gene expression patterns between the SCI + NMN group and the SCI group (SCI + NMN vs. SCI) (Figure 4A). We found that NMN (SCI + NMN vs. SCI) upregulated or downregulated many genes (Figure 4B). NMN treatment acted on mice with SCI through many classical signaling pathways related to the nervous system including the IL-17 signaling pathway, neuroactive ligand-receptor interaction, the TNF signaling pathway, cytokine-cytokine receptor interaction, and the chemokine signaling pathway (Figures 4C, D).

Further evidence from RNA-seq has shown that the biological processes involved in the response of SCI mice to NMN treatment predominantly include the response to interferon-beta, the cellular response to interferon-beta, viral defense response, and immune system processes. The cellular components that play key roles mainly include the extracellular space, the extracellular region, cell projection and neuronal projection. At the molecular level, the functional components predominantly include the structural constituents of bone, chemokine activity, peptide receptor activity, hydroxyapatite binding, and chemokine receptor binding (Figures 4E, F). Statistical results using fold change ≥ 2 and *p* < 0.05 to identify significant differences in gene expression.

In summary, our data provide novel mechanistic evidence that NMN exerts neuroprotective effects after SCI by the regulation of certain genes acting on signaling pathways related to neurological diseases. Inflammatory response, cell apoptosis, cell death, and secondary neuropathic pain after spinal cord injury hinder nerve repair and regeneration. NMN intervention may play a neuroprotective role through inflammatory response, thus delaying cell apoptosis and death.

### 3.4 Key genes and pathways of NMN in relation to nerve regeneration and neuroprotection in SCI mice

**FIGURE 5:**
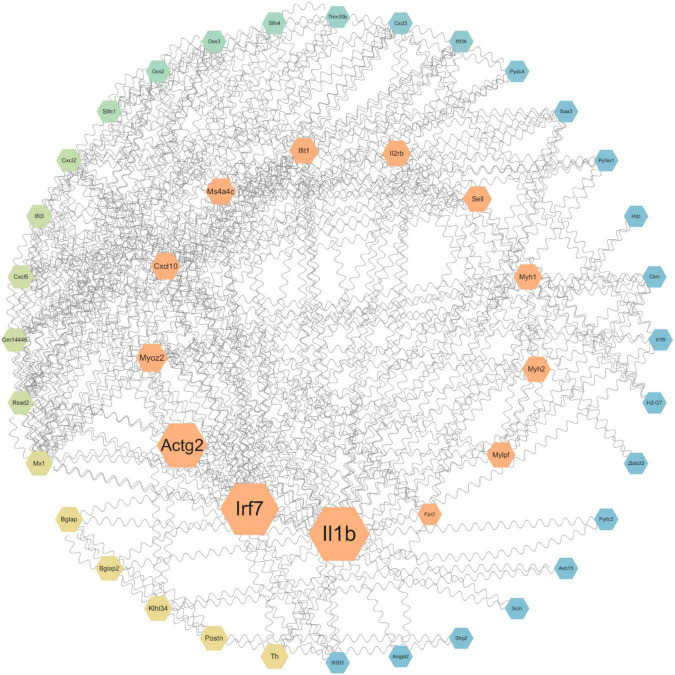
Protein-protein interaction networks showing the connection between NMN and neuroprotection after SCI. STRING and Cytoscape were used to generate a PPI network diagram for the biological interactions between DEGs identified by the RNA-seq dataset, involving immune and inflammatory functions. The size of the betweenness centrality value was used to generate a color gradient display. Molecules in the central area have the strongest association with disease. The molecules that are more dispersed to the outer circle have a smaller association with disease.

**FIGURE 6:**
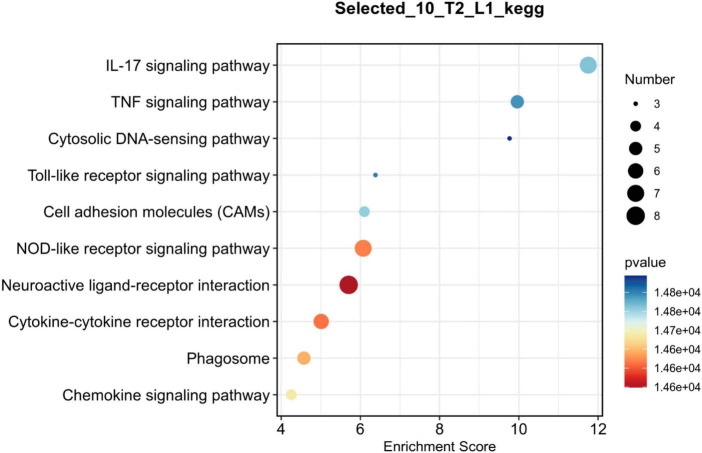
Bubble chart showing the top 10 key pathways in NMN-treated SCI mice. L1 denotes SCI group, T2 denotes SCI + NMN group. According to the size and color of the bubbles, it is possible to determine the most relevant pathways. The X-axis shows the enrichment score, and the size of the bubble area represents the number of enriched genes. The larger the bubble, the more enriched genes it contains. The color of the bubbles represents the significance of enrichment (the *p*-value). The color of the bubble changes from purple-blue-yellow-red; the smaller the *p*-value, the greater the significance. The more red the bubble color is, the smaller the *p*-value is, and the more significant the difference. The more purple the bubble color is, the larger the *p*-value is, and the less significant the difference. The Y-axis represents the names of the key pathways.

To further investigate the key genes and pathways involved in neuroprotection after SCI, we constructed protein-protein interaction (PPI) networks for key genes (Figure 5) and signaling pathways (Figure 6). The effects of NMN on SCI in mice were mainly derived from key genes, including *IL-1b*, *Irf7*, *Actg2*, *Myoz2*, *Cxcl10*, and *IL-2RB*. The key interacting pathways were the IL-17, TNF, and NOD-like receptor pathways, along with pathways associated with neuroactive ligand-receptor interactions and cytokine-cytokine receptor interactions. These results show that NMN may play an important role in SCI through cytokines (e.g., IL-1b) and chemokines (e.g., Cxcl10) through inflammation-related pathways such as the IL-17 signaling pathway and that cytokine-cytokine receptor interaction can improve the inflammatory response after SCI and play a neuroprotective role. Statistical results pathway screening was conducted according to *p* < 0.05, and *p* < 0.05 was considered statistically significant.

### 3.5 Verification of the mRNA levels of cytokines by qRT-PCR

**FIGURE 7:**
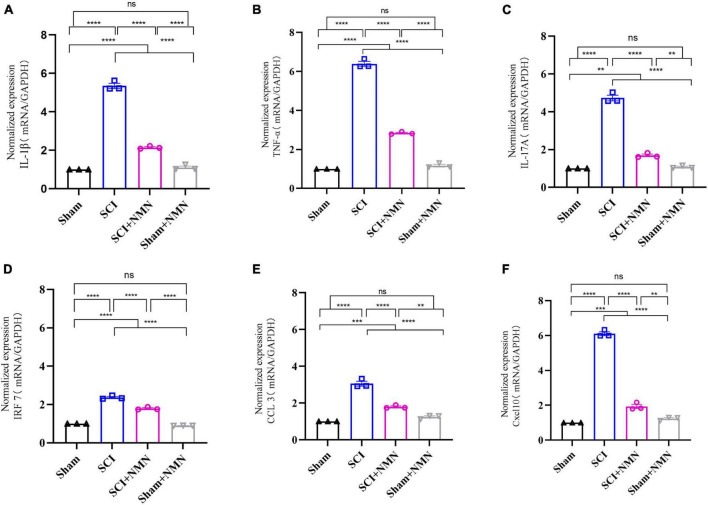
Six selected inflammation-associated factors were assessed by qRT-PCR analysis. **(A)** mRNA levels of IL-1β, *P* < 0.0001; **(B)** mRNA levels of TNF-α, *P* < 0.0001; **(C)** mRNA levels of IL-17A, *P* < 0.0001; Sham vs. SCI+NMN, *P* = 0.001; Sham+NMN vs. SCI+NMN, *P* = 0.0022; **(D)** mRNA levels of IRF7, *P* < 0.0001; **(E)** mRNA levels of CCL3, *P* < 0.0001; Sham vs. SCI+NMN, *P* = 0.0002; Sham+NMN vs. SCI+NMN, *P* = 0.0024; **(F)** mRNA levels of Cxcl10, *P* < 0.0001; Sham vs. SCI+NMN, *P* = 0.0002; Sham+NMN vs. SCI+NMN, *P* = 0.0013. Data are presented as mean SEM (*n* = 3/group). **indicate *P* < 0.01, ***indicate *P* < 0.001, ****indicate *P* < 0.0001; ns indicates no statistical difference between groups.

To validate the RNA-seq data, six genes were selected and analyzed by qRT-PCR. These genes included six DEGs (*IL-1*β, *TNF-*α, *IL-17A*, *IRF7*, *CCL3*, and *Cxcl10*) that are closely associated with inflammation. Compared with the SCI group, the mRNA expression levels of pro-inflammatory factors (*IL-1*β, *TNF-*α, and *IL-17A*) and chemokine ligand 10 (*Cxcl10*) in the spinal cord of the SCI + NMN group were significantly reduced (*p* < 0.0001). The expression levels of interferon regulatory factor 7 (*IRF7*) mRNA were also decreased (*p* < 0.0001). The levels of macrophage inflammatory protein 1-α and chemokine ligand 3 (*CCL3*) were reduced (*p* < 0.0001). The statistical results showed that compared with Sham group, mRNA expression of inflammation-related cytokines was significantly increased in SCI group and the difference was significant *p* < 0.0001. Compared with SCI group, mRNA expression of inflammation-related cytokines was significantly decreased in SCI + NMN group and the difference was significant *p* < 0.0001. The results of qRT-PCR were consistent with those of RNA-seq above, indicating that the experimental results of RNA-seq were reliable (Figure 7).

## 4 Discussion

Previous research has established NAD + depletion as a primary contributor to neuronal death in central nervous system disorders. The therapeutic efficacy of NMN as a potent precursor of NAD + in Parkinson’s disease is evident in its ability to effectively mitigate oxidative stress, cholinergic system impairment, and 5-hydroxytryptamine (5-HT) system damage induced by non-ylphenol (Huang et al., 2023). Additionally, NMN supplementation leads to an elevation in the body’s NAD + levels and an upregulation of proteins associated with the SIRT1 pathway (Li et al., 2023). The therapeutic significance of NMN in dementia is highlighted by its demonstrated efficacy in ameliorating cognitive deficits in a transgenic mouse model of Alzheimer’s disease (AD) through intraperitoneal NMN injections. This therapeutic effect is attributed to the reduction in Aβ deposition, which subsequently mitigates synaptic disruption and inflammatory responses (Wang et al., 2016; Yao et al., 2017; Poddar et al., 2019). Furthermore, NMN has been shown to significantly alter the composition of the intestinal microbiota in AD model mice by increasing the relative abundance of certain genera such as Lactobacillus and Bacteroides, which are known to produce short-chain fatty acids. This change in microbiota composition effectively ameliorates AD symptoms in the mice (Zhao et al., 2023). The improvement of white matter lesions was evidenced by an increase in the expression of myelin basic protein (MBP), along with a decrease in the intensity of SMI32 (a marker for demyelinating axons) and the SMI32/MBP ratio (Yu et al., 2022). The therapeutic efficacy of NMN in stroke is demonstrated by its ability to significantly reduce infarct size 24 h following middle cerebral artery occlusion in mice. Furthermore, no observed adverse reactions are associated with NMN treatment during the subsequent 14-day period (Zheng et al., 2023). In a collagenase-induced mouse model of intracerebral hemorrhage (ICH), NMN treatment was administered to mice 30 min post-ICH. The findings demonstrated that NMN effectively reduced the expression of intercellular adhesion molecule-1 (ICAM-1), suppressed microglial activation, and attenuated neutrophil infiltration. This effect was mediated by the upregulation of two cytoprotective proteins, heme oxygenase-1 (HO-1) and nuclear factor erythrocyte 2-associated factor 2 (Nrf2) (Wei et al., 2017b). NMN treatment significantly mitigated brain edema, neuronal apoptosis, oxidative stress, and neuroinflammation in the mice, thereby exerting a neuroprotective effect on cerebral hemorrhage. Considering the significant research conducted on NMN in the context of neurological disorders, we postulate that NMN holds substantial potential for neuroprotection. However, the mechanism by which NMN operates in the context of spinal cord injury remains largely unexplored, underscoring the necessity and significance of our present study.

In this study, we systematically investigated the mechanisms underlying the neuroprotective effects of nicotinamide mononucleotide (NMN) in SCI by RNA-seq technology (Chen et al., 2013; Sharif-Alhoseini et al., 2017). We found that SCI mice treated with NMN had improved motor function and increased thresholds for secondary neuropathic pain after injury. We found that NMN alleviated progression of the inflammatory response after SCI by reducing the expression of pro-inflammatory factors and chemokines, improved neuropathic pain in SCI mice, and exerted a neuroprotective effect to improve motor function. We also found that certain signaling pathways were regulated by NMN after SCI, including the IL-17, TNF, cytokine-cytokine receptor interaction, and chemokine signaling pathways.

A large number of studies have been conducted, which are related to the pathways of inflammatory response after spinal cord injury. Neuroactive ligand-receptor interaction is enriched in the gene regulatory pathways associated with mRNA-lncRNA co-expression patterns after SCI in rats and that this may be related to neural repair after injury (Liu W. et al., 2022). The TNF signaling pathway is considered to play an important role in the rescue of neuronal apoptosis after SCI (Zhang et al., 2018). The NF-kappa B signaling pathway (Fei et al., 2021; Liang et al., 2022) and the MAPK signaling pathway (Liu et al., 2020; Liu C. et al., 2022), as classical inflammation-related pathways, are known to play an important role in neuroinflammatory response after SCI. The chemokine signaling pathway (the CXCL12/CXCR4 signaling pathway) is also known to play a role in the occurrence and maintenance of neuropathic pain after SCI (Liu Z. Y. et al., 2019). The Toll-like receptor signaling pathway is involved in the post-activation inhibition of glial cells and inflammatory response in mice with peripheral nerve injury to reduce secondary injury (Wei et al., 2022). Relevant studies have reported that IL-17A plays an important role in the progression of diseases in addition to participating in the inflammatory response (Komiyama et al., 2006; Oh et al., 2016). IL-17A is involved in regulating the proliferation of neural stem cells both *in vivo* and *in vitro* (Li et al., 2013; Liu et al., 2014) and may be involved in regulating the pathogenesis of SCI. Reduced IL-17A levels may promote recovery of motor function after spinal cord injury (Hill et al., 2011). Cytokine-cytokine receptor interaction and cell cycle regulation (Li Z. et al., 2020). Several chemokines are upregulated after peripheral nerve injury and are involved in the pathogenesis of neuropathic pain through different forms of neuron-glial interactions in the spinal cord; for example, the chemokine CX3CL1 induces microglial activation *via* the microglial receptor CX3CR1 while spinal astrocytes are activated by CXCR5. Chemokines CCL2 and CXCL1 increase excitatory synaptic transmission *via* CCR2 and CXCR2 in spinal cord neurons (Zhang et al., 2017).

To comprehensively and systematically investigate the mechanisms of action underlying the effect of NMN on SCI, we performed in-depth RNA-seq. Our RNA-seq data provide significant information about RNA expression profiles, thus allowing us to identify specific molecular mechanisms and previously overlooked genes and pathways (Wolfien et al., 2016). By performing RNA-seq and functional enrichment analysis, we found that neuronal apoptosis and death after SCI, and the inflammatory response after SCI was effectively ameliorated by NMN. No previous study has reported whether NMN can play a neuroprotective role by reducing the inflammatory response and relieving neuropathic pain and promoting the recovery of motor function mice. An increasing body of evidence now shows that the inflammatory response after SCI can be triggered by a variety of signals, such as proinflammatory factors, chemokines, and other cytokines (Campbell et al., 2008; Tsarouchas et al., 2018; Li H. L. et al., 2020; Lin et al., 2021; Wang et al., 2021). This was also supported by the results of our qRT-PCR analysis, which suggested that NMN may play a neuroprotective role by reducing inflammatory response and inhibiting the release of proinflammatory cytokines and chemokines to promote nerve regeneration and repair. In this study, qRT-PCR results showed that the expression of inflammatory factors and chemokines at the site of injury increased after SCI, and that the expression levels of related indicators at the site of injury were reduced in the SCI + NMN group. In animal and cellular models, NMN administration has been found to improve the senescence of retinal pigment epithelium (RPE) by reducing DNA damage and maintaining mitochondrial function (Ren et al., 2022). NMN treatment affects NAD + metabolism and also affects the transformation of macrophage phenotype. The secretion of inflammatory mediators and proinflammatory activation in the macrophages of NMN-treated mice was reduced; this plays a role in reducing the inflammatory response by reprogramming peritoneal macrophages to anti-inflammatory/pro-inflammatory phenotypes (Cros et al., 2022). NMN has also been shown to reduce lipopolysaccharide (LPS)-induced macrophage inflammation and oxidative stress by reducing the expression of cyclooxygenase-2 (COX-2) in macrophages (Xie et al., 2017; Liu et al., 2021). NMN can also improve STRT1 function and cause alterations in macrophage phagocytosis and M1 polarization *via* the SIRT1, IL-17A, and Notch pathways (Meng et al., 2021). Other studies have shown that NMN exerts neuroprotective effects by inhibiting neuronal apoptosis and death, delaying neuroinflammation, affecting macrophage polarization, and protecting the integrity of the blood-brain barrier (Kitaoka et al., 2009; Wei et al., 2017a,b; Harlan et al., 2019; Chen et al., 2020; Chini et al., 2022). SIRT2 is essential for LPS-induced microglial cell activation, and inhibiting SIRT2 can reduce microglial cell activation, alleviate neuroinflammation, decrease dopamine neuronal death, and delay Parkinson’s disease neuroinflammation. This effect may be related to SIRT2 activating NF-κB transcription to promote inflammation and neurocellular death (Liu Y. et al., 2019). Concurrently, NMN treatment activates the antioxidant pathways (Nrf2 and Hmox-1), and it has been confirmed by other researchers that NMN treatment exerts a protective effect against oxidative stress-induced cell death (Lee et al., 2022). In light of our research findings, it is observed that the NAD + content in spinal cord tissue significantly decreases following spinal cord injury, accompanied by alterations in the expression levels of the NAD + -dependent enzyme genes, such as SIRT2 and Hmox-1. This suggests that both SIRT2 and Hmox-1 may serve as key molecules for the neuroprotective effects of NMN in spinal cord injury diseases. Although NMN may represent a potential candidate for the treatment of SCI by reducing the inflammatory response, the specific molecular mechanisms by which NMN exerts neuroprotective effects still need to be investigated further before it can be used as a drug.

In this study, we examine NMN, the precursor of NAD + . Another NAD + precursor, nicotinamide riboside (NR), that has been extensively explored by researchers. NR is initially converted to nicotinamide mononucleotide (NMN) *in vivo* via the action of nicotinamide ribokinase (NRK), ultimately giving rise to NAD + . Subsequently, NMN is converted to NAD + by niacinamide mononucleotide transferase (Yoshino et al., 2018). NR is thought to be effective in increasing NAD + levels in tissues such as muscle and liver, and NMN is thought to play a crucial role in cellular energy metabolism and DNA repair (Braidy et al., 2019; Reiten et al., 2021). Some studies have shown that patients with Alzheimer’s disease can increase their NAD + levels by NR supplementation as this leads to reduced expression levels of proinflammatory cytokines and the NLRP3 inflammasome; NR supplementation also helps to reduce DNA damage, apoptosis and cellular senescence. Thus, NR reduces the activation of microglia and astrocytes and plays an important role in inflammation-related diseases (Hou et al., 2021; Roboon et al., 2021). Relative to NR, NMN is more direct in terms of NAD + conversion. Compared with NAD+, NMN has a smaller molecular weight and can be efficiently absorbed and utilized. Because it can be administered in a range of different ways and has a high clinical conversion rate, NMN has gradually replaced direct NAD + supplements. NMN has a small molecular weight and plays an important role in the pathogenesis of various diseases as an effective precursor to increase NAD + levels (Stein and Imai, 2014; Yoon et al., 2015; Mills et al., 2016). Given the observed effects associated with NR, it is imperative to delve into the precise mechanism of action of NR and compare it to that of NMN. Such an investigation would greatly facilitate the selection and dosage control of therapeutic drugs. To comprehensively explore the molecular mechanisms of NMN, particularly those involving microRNA, circRNA, and lncRNA, additional *in vivo* and *in vitro* experiments are warranted to elucidate crucial targets. This endeavor will enhance our comprehension of the genuine clinical efficacy of NMN in spinal cord injury (SCI) treatment and establish a theoretical foundation for the clinical translation and application of NMN.

## 5 Conclusion

Our analysis demonstrated that NMN restored motor function and relieved pain in mice by supplementing NAD + levels after SCI. RNA-seq and qRT-PCR analysis confirmed that NMN down-regulates the expression of inflammatory factors and chemokines, and exerted influence on the IL-17, TNF, Toll-like receptor signaling, NOD-like receptor signaling, and chemokine signaling pathways.

## Data availability statement

The data presented in the study are deposited in the NCBI Sequence Read Archive (SRA) repository, accession number BioProject: PRJNA977966, https://dataview.ncbi.nlm.nih.gov/object/PRJNA977966?reviewer=oe2hm4h1grodc6teme3vl46std.

## Ethics statement

The animal protocol was approved by the Animal Care and Use Committee of Capital Medical University (Ethics Batch No. AEEI-2023-104). The study was conducted in accordance with the local legislation and institutional requirements.

## Author contributions

CZ: Data curation, Formal analysis, Investigation, Methodology, Project administration, Resources, Software, Validation, Visualization, Writing—original draft. YL: Data curation, Investigation, Methodology, Software, Validation, Writing—review and editing. FB: Methodology, Validation, Writing—review and editing. ZT: Formal analysis, Writing—review and editing. HK: Validation, Writing—review and editing. XX: Formal analysis, Writing—review and editing. ZL: Investigation, Writing—review and editing. WL: Formal analysis, Validation, Writing—review and editing. YP: Formal analysis, Writing—review and editing. FG: Writing—review and editing. DY: Writing—review and editing. XW: Formal analysis, Writing—review and editing. HD: Formal analysis, Writing—review and editing. SG: Investigation, Writing—review and editing. HG: Formal analysis, Writing—review and editing. LD: Writing—review and editing. YY: Conceptualization, Funding acquisition, Methodology, Project administration, Resource, Supervision, Validation, Writing—review and editing. JL: Conceptualization, Funding acquisition, Methodology, Project administration, Resources, Supervision, Visualization, Writing—review and editing.

## Footnotes

1
https://www.ncbi.nlm.nih.gov/

2
https://www.psc.edu/resources/software/hisat-2/

3
https://cn.string-db.org/
