# Supplementary material for: The identification of new roles for nicotinamide mononucleotide after spinal cord injury in mice: an RNA-seq and global gene expression study
Source: Front Cell Neurosci. 2023 Dec 14;17:1323566. doi: 10.3389/fncel.2023.1323566 (PMC10752985; doi:10.3389/fncel.2023.1323566)

**Supplementary Table 1. Primer sequences of qRT-PCR test.**

| **Items** | **Primer (5’→3’)** |
| --- | --- |
| IL-1β | Forward: TCGACAATATGGCAATCTAG |
|  | Reverse: GGATGGTGACACTATAGGA |
| TNF-α | Forward: TTATGTGACCACAGCAATGTAGGA |
|  | Reverse: CCCAGTGTGTGGCCATATTCTTA |
| IL-17A | Forward: TTTAACTCCCTTGGCGCAAAA |
|  | Reverse: CTTTCCCTCCGCATTGACAC |
| CCL3 | Forward: CACTCGCAGTTGTGGTTGAT |
|  | Reverse: CTCCCAGTAAAGGCTTCTCC |
| IRF7 | Forward: AAGACAAGGCGAGATGCAT |
|  | Reverse: CTGCAAGAGCGGGATGACG |
| Cxcl10 | Forward: CCAAGTGCTGCCGTCATTTTC |
|  | Reverse: GGCTCGCAGGGATGATTTCAA |
| GAPDH | Forward: GCTAGAAGGCTGGGCTGGCTTG |
|  | Reverse: AGGGTGGCGACATCCACAC |

**Supplementary Figure 1. Amplification plots for these candidate inflammatory factors and GAPDH.** （A）GAPDH. （B）IL-1β. （C）TNF-α. （D）IL-17A. （E）IRF-7. （F）CCL-3. （G）Cxcl10.


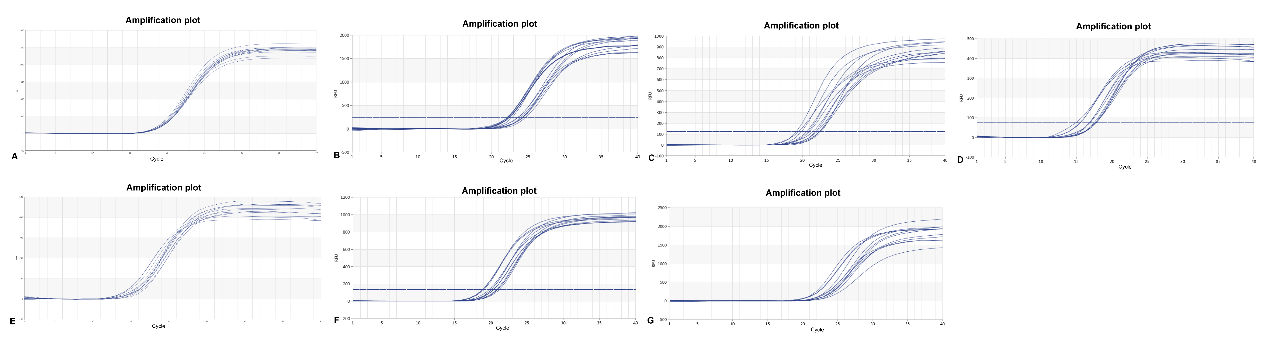


**Supplementary Figure 2. Melt curve plots for these candidate inflammatory factors and GAPDH.** （A） GAPDH. （B）IL-1β. （C）TNF-α. （D）IL-17A. （E）IRF-7. （F）CCL-3. （G）Cxcl10.


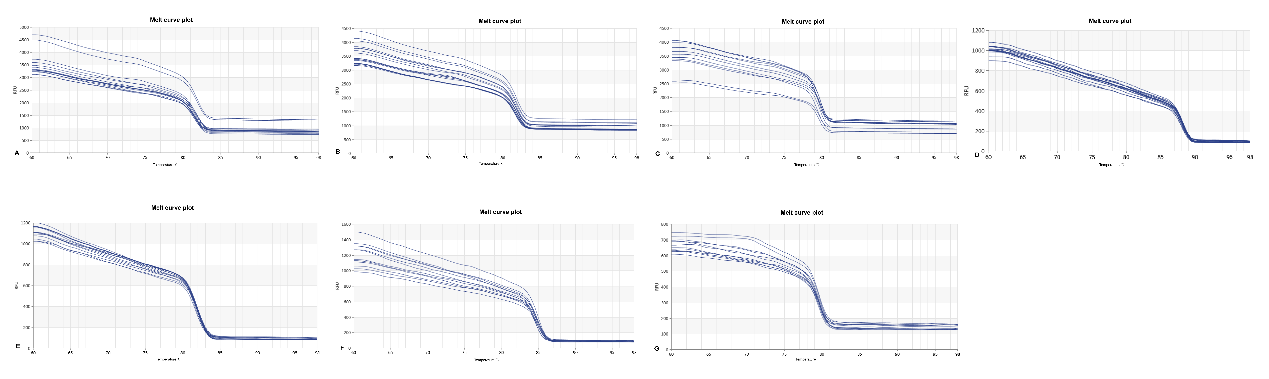


**Supplementary Figure 3. Melt peak curve plots for these candidate inflammatory factors and GAPDH.** （A） GAPDH. （B）IL-1β. （C）TNF-α. （D）IL-17A. （E）IRF-7. （F）CCL-3. （G）Cxcl10.


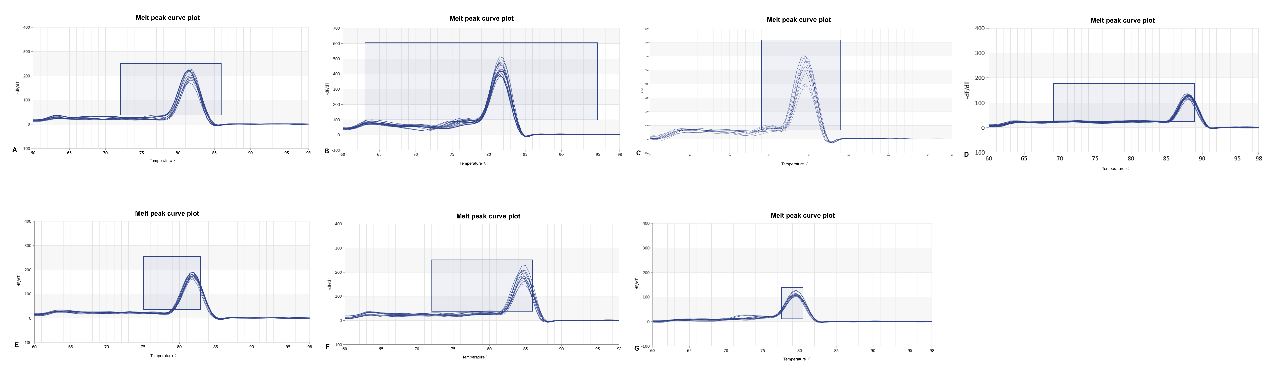

Supplement: Supplementary file 1 [file Data_Sheet_1.docx]
